# A new method for assessing tibial torsion using computerized tomography in a pediatric population

**DOI:** 10.3389/fped.2024.1368820

**Published:** 2024-07-15

**Authors:** Nathaly Gavira, Blaise Cochard, Nastassia Guanziroli, Giorgio Di Laura Frattura, Romain Dayer, Dimitri Ceroni

**Affiliations:** ^1^ Pediatric Orthopedics Unit, Pediatric Surgery Service, University Hospitals of Geneva, Geneva, Switzerland; ^2^ Division of Orthopedics and Trauma Surgery, University Hospitals of Geneva, Geneva, Switzerland

**Keywords:** pediatric tibial torsion, scanner assessment, talar angle, intermalleolar angle, talar axis

## Abstract

**Purpose:**

Tibial torsion disorders may lead to abnormal gait, frequently leading to a consultation with a pediatric orthopedic surgeon. The present study evaluated an alternative method for assessing tibial torsion on computerized tomography (CT) images that considers the tibial distal axis to be equivalent to the geometric axis of the tibiotalar joint.

**Methods:**

One hundred CT scans were reviewed retrospectively, and four measurements were taken: proximal transtibial angle (PTTA), posterior margin tibial plateau angle (PMTPA), intermalleolar angle (IMA), and talar angle (TA). The tibial torsion angle was then calculated using these different angles.

**Results:**

The patient cohort comprised 38 girls and 62 boys, with a mean age of 12 ± 4.4 years. Median PTTA and PMTPA were −8.4° ± 14.7° and −8.8° ± 14.2°, respectively, with no statistically significant difference. Mean IMA and TA were 23° ± 16.2° and 17.2° ± 16.9°, respectively, with a statistically significant difference. Mean total measurement time per patient was 6'44", with means of 2'24" for PTTA, 36" for PMTPA, 2'14" for IMA, and 1'12" for TA.

**Conclusion:**

Tibial torsion values may differ significantly depending on the axis chosen to define tibial orientation. At the level of the proximal tibia, the choice of PTTA or PMTPA had little influence on the calculation of the tibial torsion angle. There was a significant difference of 5.8° when measuring the distal tibia. Measuring the PMTPA and TA is probably more suited for use in clinical practice because their tracing is simple and faster.

## 1 Introduction

Torsional abnormalities of the lower limbs frequently lead to consultations with pediatric orthopedic surgeons due to parental concerns about the development of walking and cosmetic issues and their social repercussions (1). In addition to these aspects, torsional alterations of the lower limbs impact the biomechanics of gait (2, 3), with clinically visible disturbances of the foot progression angle. The latter is defined as the angle between the line passing through the heel and the second metatarsal and the line of gait progression during the stance phase (4). This value is mainly influenced by femoral and tibial torsion (5).

Several techniques for quantifying torsional disorders of the tibia have been described. Although some suggest assessment using biplanar radiography or magnetic resonance imaging (6), computed tomography (CT) imaging is currently considered the gold standard (7). CT's advantages are its widespread availability, proven accuracy, short examination time, and cost-effectiveness (8). As noted, there is a lack of standardization regarding current tibial torsion (TT) measurement techniques (9–11). TT measurements can be made by comparing the axis of the proximal tibia (12), taking either the transtibial axis (9) or the posterior margin of the tibial plateau line (10) as a reference, with the intermalleolar axis (11, 13). These measurements are sub-optimal, however; firstly because of significant inter- and intra-observer variability (8), and secondly because the foot's orientation is determined more by the orientation of the tibiofibular mortise and the shape of the talus than by the alignment of the two malleoli.

The present study sought to optimize the assessment of tibial torsion by evaluating and comparing the different techniques intended for its measurement. The study's secondary purpose was to investigate the feasibility of using an alternative method for determining distal tibial orientation, based on the talar axis instead of the intermalleolar axis, as the talar axis corresponds to the geometric axis of the tibiotalar joint and correlates more adequately with foot position during gait (14). The study hypothesis was that measurements based on the intermalleolar axis and those based on the talar axis would be significantly different**.**

## 2 Materials and methods

### 2.1 Study design and participants

After approval by the Children's Hospital's Review Board (Swiss Research Ethics Committee: Project ID 2023-00508), the medical charts of 120 consecutive patients (age greater than 5 years old) with available CT imaging realized for suspected torsional disorders between January 2015 to December 2022 were retrospectively analyzed and investigated for tibial torsion. Detailed demographic data were collected from patients’ electronic health records, including sex and age at the time of torsion measurement. Exclusion criteria were being above 18 years old (legally adults), neurological or syndromic pathologies, a history of proximal tibia or ankle fracture, malformations such as clubfoot, coalition, or equinus, the presence of metal implants within the scanning field, and low-quality CT images. In our institution,on average, 12 children/year received lower limb CT for suspected torsionnal disorders.

### 2.2 Technical parameters of the CT protocol for tibial torsion measurement

Participants had undergone a CT of their lower limbs to explore torsional disorders while lying supine with their feet secured together to avoid movement. All the scans were performed using the same scanner (Siemens Somaton Definition Edge number 10430603/73165, Philips Medical Systems Inc., Cleveland, OH, USA) with the same scanning parameters. The CT protocol used helical acquisition for its speed and to reduce measurement errors due to patient movements. Radiologists had followed our institution's standard operating procedures, which emphasize using the minimum scan length necessary to completely delineate the relevant anatomical structures based on prominent anatomical landmarks in the anteroposterior scout view (cuts are made at the level of the hips, knees and ankles). The CT scan used a nominal single collimation width of 0.6 mm, with a total collimation width of 38.4 mm at 80 kV. The knee acquisition protocol was: exposure time 4.24’’, scanning length 261 mm, exposure range 237 mm at 58–79 mA, exposure time per rotation 0.5’’, and 0.52 mGy. The ankle acquisition protocol was: exposure time 2.69’’, scanning length 165 mm, exposure range 141 mm at 49–59 mA, exposure time per rotation 0.5’’, and 0.44 mGy.

### 2.3 Feasibility evaluation

Each tibial measurement was performed independently by two pediatric orthopedists—one senior orthopedist with 25 years of experience and another with 5 years of experience in musculoskeletal pathologies.

Levels of inter- and intra-observer agreement were analyzed using an intraclass correlation coefficient (ICC), with the resulting values considered poorly reliable (<0.5), moderately reliable (0.5–0.75), of good reliability (>0.75–0.9), or excellent (>0.9). Levels of intra-observer agreement were evaluated after the younger orthopedic surgeon had measured 22 CT scan images twice at least 24 h apart and with other CT scan images measured in between. The level of inter-observer agreement was calculated by comparing the measurements of 44 CT scan images made by the highly experienced orthopedist and the younger orthopedist. Tests were evaluated using a significance level of *p* < 0.05.

### 2.4 Definition of axis and measured angles

**Figure 1:**
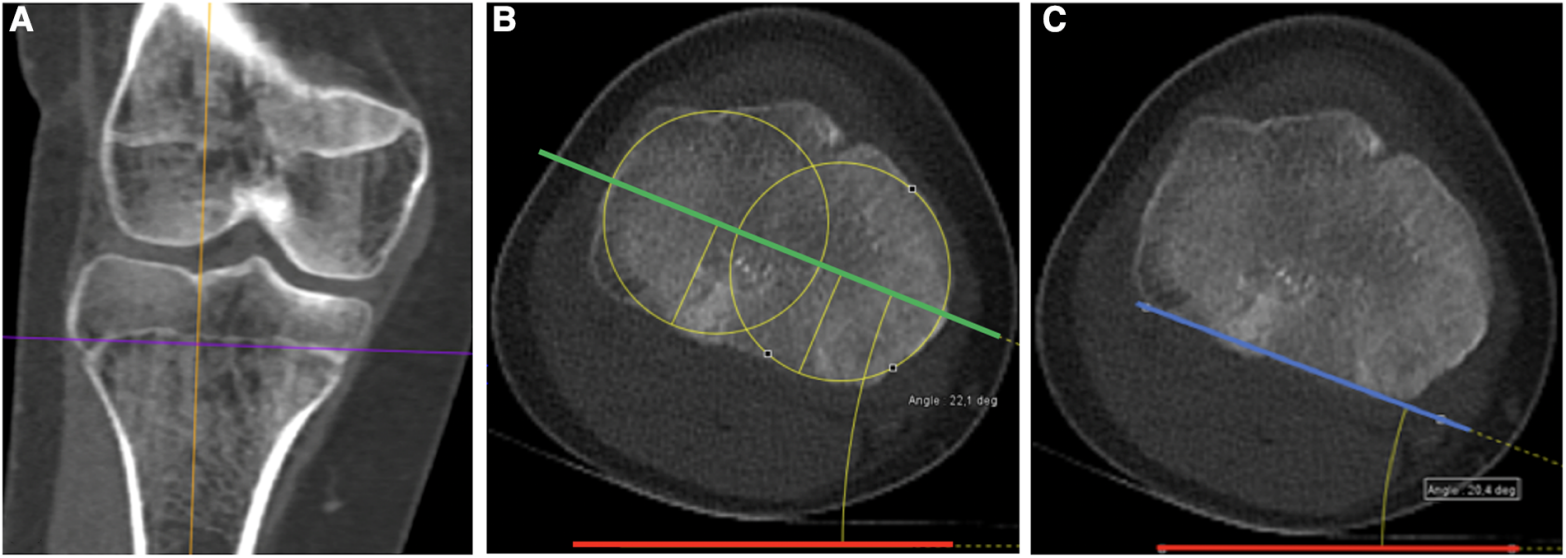
Axis definition at the proximal tibia. (**A**) Coronal CT scan view of the right knee. Analysis is performed on an axial section located at the physeal level (purple line). (**B**) Corresponding axial view illustrating the proximal transtibial angle (PTTA) measurement. PTTA is defined as the angle between the horizontal (red line) and the proximal transtibial axis (green line), defined as the center of the two circles of the medial and lateral tibial plateau (yellow circles). (**C**) Corresponding axial view illustrating the posterior margin tibial plateau angle (PMTPA) measurement, which is defined as the angle between the horizontal (red line) and the posterior margin of the tibial plateau (blue line).

The proximal tibial axis was drawn using two recognized techniques (Figure 1). Measurements were performed on an axial section located at the level of the growth plate. Two axes were used at the level of the proximal tibia, i.e., the transtibial axis and the line posterior to the margin of the tibial plateau. The first angle was drawn and measured between the horizontal line and the proximal transtibial axis, and this was named the proximal transtibial angle (PTTA) (9). The second angle was drawn and measured between the horizontal line and the line along the posterior margin of the tibial proximal plateau, and this was named the posterior margin tibial proximal angle (PMTPA) (10).

When the tibial axis was internally rotated with respect to the horizontal, we assigned a negative value to the angle; when the tibial axis was externally rotated with respect to the horizontal, we assigned a positive value.

**Figure 2:**
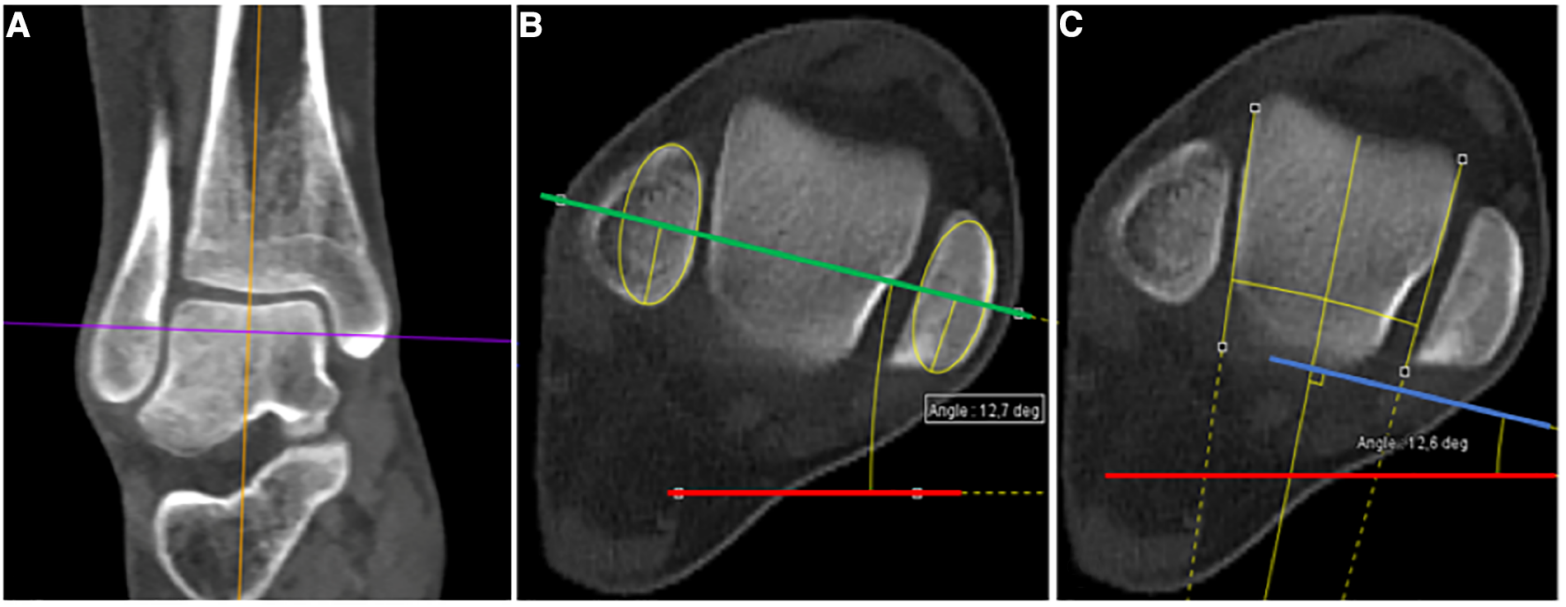
Angle measurements at the ankle. (**A**) Coronal CT scan view of the right ankle. Analysis is performed on the first axial section passing through the body of the talus, the distal fibular epiphysis and the medial malleolus (purple line). (**B**) Corresponding axial view illustrating the intermalleolar angle (IMA) measurement. IMA is defined as the angle between the horizontal (red line) and a central line through the tibial and fibular malleoli (green line). (**C**) Corresponding axial view illustrating the talar angle (TA) measurement. TA is defined as the angle between the horizontal (red line) and the orientation of the talus axis in the tibiofibular mortise (blue line). Its orientation is determined by tracing the tangent to the internal and external surfaces of the talus. Next, a bisector of the two tangents is drawn, and finally, a line perpendicular to this bisector is drawn (blue line).

Distally, analyses were performed on the first axial section passing through the body of the talus, the distal fibular epiphysis, and the medial malleolus. The ankle's orientation was based on the intermalleolar angle (IMA) measured between the horizontal line and a central line through the tibial and fibular malleoli of the ankle joint, i.e., the intermalleolar axis (11, 13) (Figure 2). In addition to this measurement, we developed an alternative method of estimating the tibial axis at the ankle by considering the position of the talus in the tibiotalar joint (named the talar axis in this study). The orientation of the talus in the tibiofibular mortise was calculated by tracing the tangent to the internal and external surfaces of the talus. Next, a bisector of the two tangents was drawn, and a line perpendicular to this bisector was designated the talar axis, closer to the geometric axis of the tibiotalar joint. The talar angle (TA) at the ankle was then drawn between the horizontal line and the line described immediately above (Figure 2).

In the same way, we assigned a negative value to the measurement of the ankle’s angle when it was internally rotated with respect to the horizontal, and we assigned it a positive value when it was externally rotated with respect to the horizontal.

### 2.5 Statistical analyses

Statistical analyses were performed using the JMP software package (Version 16.2.0, SAS Institute, Cary, NC, USA). The Shapiro–Wilk W test was performed to verify the normality of continuous variables. Normally distributed variables are reported as an arithmetic mean and standard deviation. Non-normally distributed variables are reported as a median with a total range. If the differences between the variables are normally distributed, a Student t-test was applied. If not, a Wilcoxon *t*-test was performed. Correlated ordinal variables were analyzed using the Pearson correlation coefficient.

## 3 Results

### 3.1 Population

**Figure 3:**
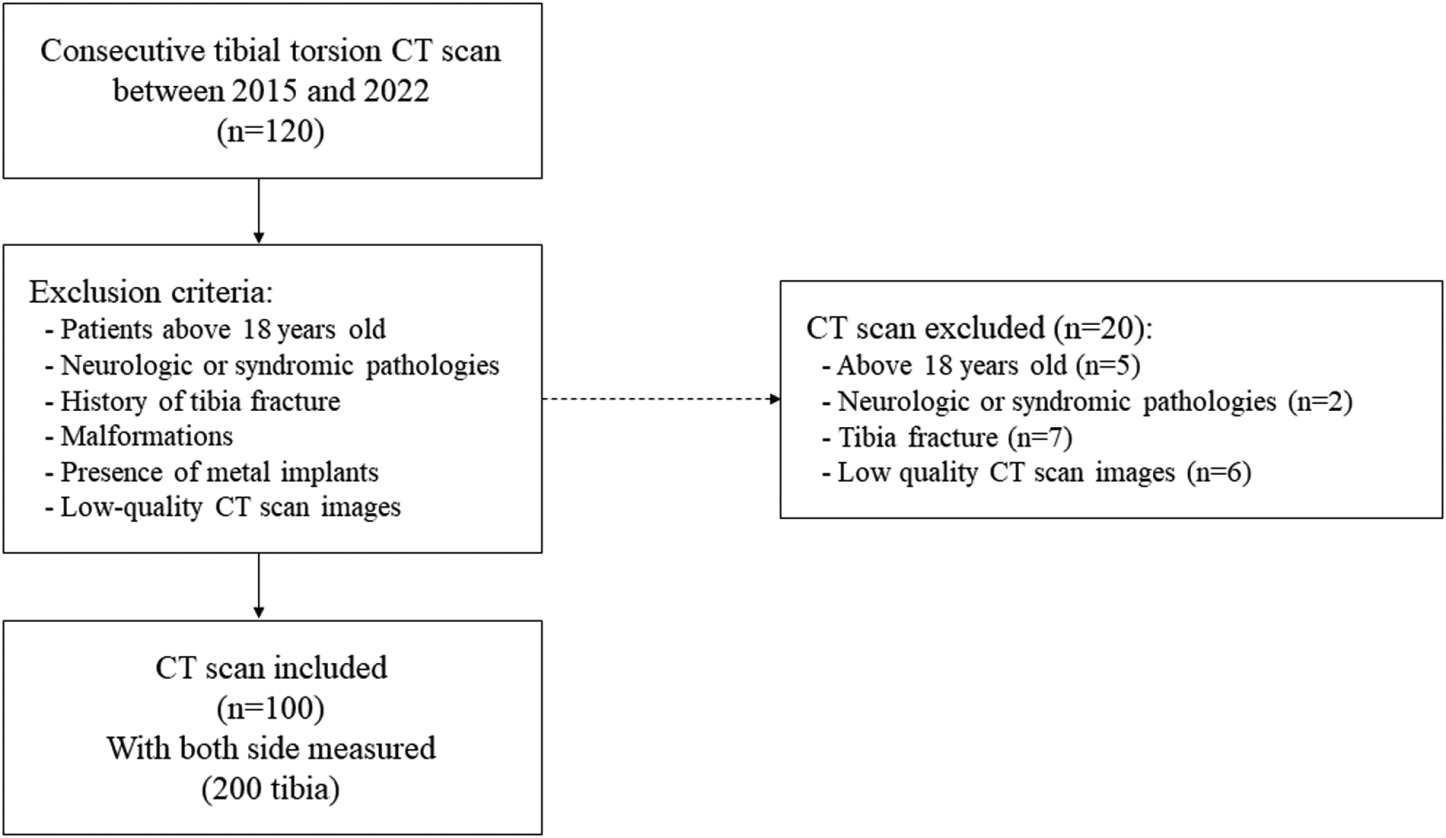
Flowchart describing the inclusion and exclusion process.

A total of 100 complete electronic health records with useable, good-quality CT scan images were included in the study after applying our exclusion criteria. The term good quality reflects the fact that the CT scanner has sufficient sections at the level of interest, and that these are perfectly perpendicular to the longitudinal axis of the bone investigated. Tibial measurements were performed on both sides of each patient, with a total of 200 tibias analyzed as shown in the flow chart (Figure 3). The patient group included 38 girls and 62 boys. The overall mean age was 12.0 ± 4.4 years (range: 5–18 years), with means of 12.0 ± 4.2 years for girls and 12.3 ± 3.6 years for boys. This study having been carried out in a pediatric hospital, only participants aged under 18 could be included.

### 3.2 Proximal tibia measurements

The proximal transtibial and the posterior margin of the tibial proximal plateau angles’ variables were not normally distributed, with *p* = 0.01 and *p* < 0.01 respectively (Table 1). The median value for the PTTA was −8.4° [−43.2°; 45.9°] and the median value for the PMTPA was −8.8° [−38.4°; 46.0°]. There was no statistically significant difference (*p* = 0.09) between these two measures.

**Table 1:** Angle measurement distributions.

	PTTA	PMTPA	IMA	TA
*N*	200	200	200	200
Missing	0	0	0	0
Mean	−7.1°	−7.6°	23.0°	17.2°
Median	−8.4°	−8.8°	24.1°	18.3°
Standard deviation	14.7°	14.2°	16.2°	16.9°
Minimum	−43.2°	−38.4°	−18.7°	−31.5°
Maximum	45.9°	46.0°	78.8°	73.9°

PTTA, proximal transtibial angle; PMTPA, posterior margin tibial plateau angle; IMA, intermalleolar angle; TA, talar angle.

**Figure 4:**
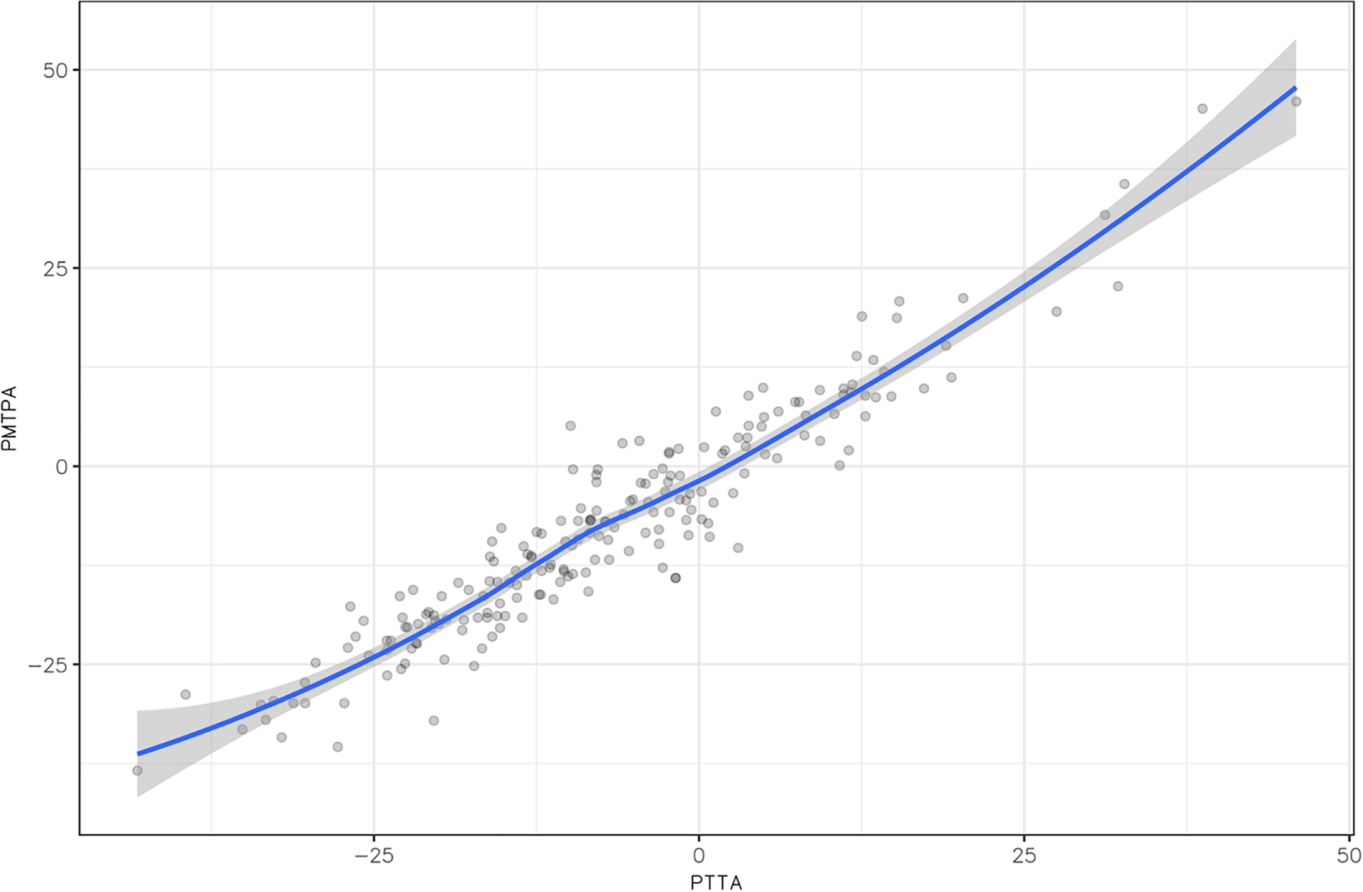
Correlation matrix between the proximal transtibial angle (PTTA) and the posterior margin tibial plateau angle (PMTPA), demonstrating an excellent correlation (*p* < 0.001).

The correlation between the PTTA and PMTPA measurements was estimated at 0.95 [95% CI (0.9361, 0.9631); *p* < 0.01], which is considered excellent (Figure 4). For intra-observer agreement, the ICC was 0.98 [95% CI (0.95, 0.99)] for the PTTA and 0.92 [95% CI (0.81, 0.97)] for the PMTPA. For inter-observer agreement, the ICC was 0.94 [95% CI (0.90, 0.97)] for the PTTA and 0.93 [95% CI (0.87, 0.96)] for the PMTPA. Since there were no statistically significant differences in the proximal tibial angles defined by the two techniques, we retained the simplest measurement to perform, namely the PMPTA, for subsequently calculating the tibial torsional angle.

### 3.3 Distal tibial measurements at the ankle level

**Figure 5:**
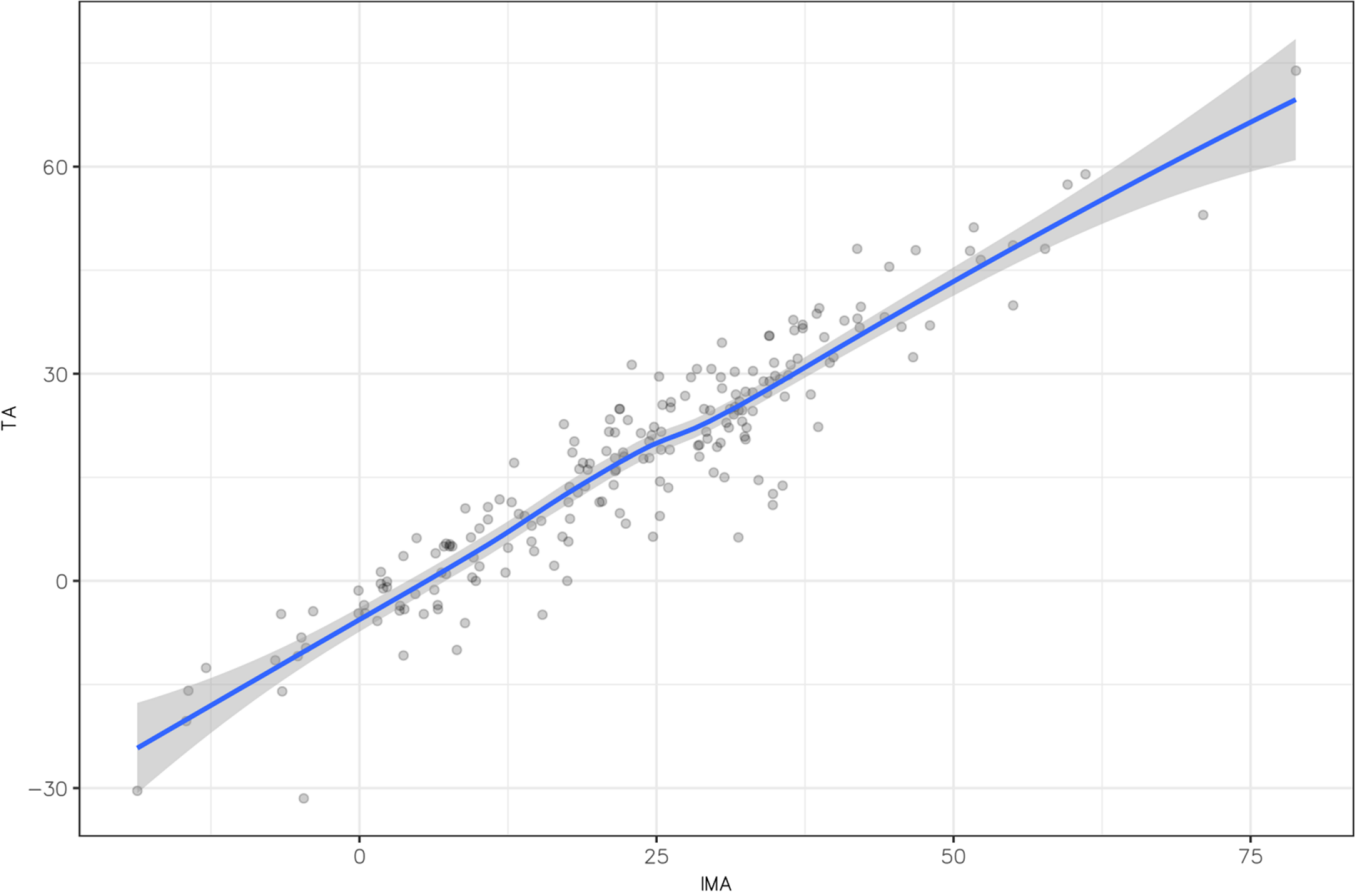
Correlation matrix between intermalleolar angle (IMA) and talar angle (TA), demonstrating an excellent correlation (*p* < 0.001).

The IMA and TA variables were normally distributed, with *p* = 0.16 and *p* = 0.67 respectively (Table 1). The mean IMA was 5.8° greater than the TA, at 23.0° ± 16.2° vs. 17.2° ± 16.9°, respectively, with a statistically significant difference (*p* < 0.001). The correlation between the IMA and TA measurements was estimated at 0.94 [95% CI (0.92, 0.95); *p* < 0.001] (Figure 5), which is considered excellent. For intra-observer agreement, the ICC was 0.999 (95% CI [0.996, 0.999] for the IMA and 0.99 [95% CI (0.98, 0.997)] for the TA. For inter-observer agreement, the ICC was 0.95 [95% CI (0.91, 0.97)] for the IMA and 0.95 [95% CI (0.9, 0.97)] for the TA.

### 3.4 Time required for measurement

**Figure 6:**
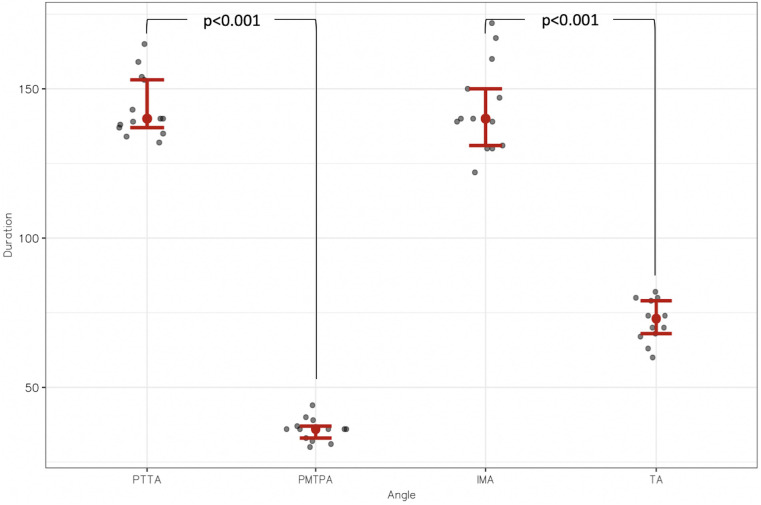
Distribution of measurement durations in seconds (s) between proximal transtibial angle (PTTA) and posterior margin tibial plateau angle (PMTPA); and between intermalleolar angle (IMA) and talar angle (TA).

The mean total time required to perform these four measurements taken from CT images was 404 ± 35 s (6 min 44 s ± 35 s), with means of 144 ± 11 s (2 min 24 s ± 11 s) for the PTTA, 36 ± 4 s for the PMTPA, 134 ± 15 s (2 min 14 s ± 15 s) for the IMA, and 72 ± 7 s (1 min 12 s ± 7 s) for the TA. The PMTPA measurement was significantly faster than the PTTA measurement of 108 s (*p* < 0.001). The TA measurement was significantly faster than the IMA measurement of 62 s (*p* < 0.001) (Figure 6).

### 3.5 Tibial torsion angle calculation

Considering the above results, two tibial torsion angles were designated as follows:

• Tibial torsion angle 1 (TTA1), when the posterior margin tibial plateau axis and intermalleolar axis were used to calculate the angle.
• Tibial torsion angle 2 (TTA2), when the posterior margin tibial plateau axis and talar axis were used to calculate the angle.

The mean tibial torsion angles were 30.5° ± 15.1° for TTA1 and 24.6° ± 16.5° for TTA2. The mean times required to measure the entire tibial torsion were 2'50" ± 19" for TT1 and 1'48" ± 11" for TT2.

## 4 Discussion

The present study aimed to optimize the assessment of tibial torsion by evaluating and comparing the different existing techniques for measuring it. The study's secondary purpose was to investigate the feasibility of an alternative method for determining distal tibial orientation, based on the talar axis instead of the intermalleolar angle, as the former corresponds to the geometric axis of the tibiotalar joint and correlates more adequately with the foot's position during gait (14).

Firstly, our results highlighted that the two axes defined to describe the proximal tibia's orientation do not constitute a factor of variability in the measurement of tibial torsion. Indeed, using either the transtibial axis or the line along the posterior margin of the tibial plateau angle does not interfere with these measurements, with the two techniques demonstrating excellent correlation and reproducibility. This led us to think that the definition of proximal tibial axis is not the problem with tibial torsion measurements. Many authors have hypothesized that there was great variability in the proximal tibial epiphysis, making it hard to determine the proximal tibia’s axis in adults (15–17). Instead, our results suggest that the definition and tracing of this tibial torsion angle line is not subject to substantial variations, regardless of the two techniques used (PTTA and PMTPA), and that the measurement is both consistent and reproducible if performed on an axial section located at the growth plate level in children.

In contrast to the femur, determining the ankle joint axis to the tibia is less well-defined (9, 11–13). On this point, it is recognized that this uncertainty essentially rests on the distal reference framework, which is based on the intermalleolar angle. This angle is highly dependent on the distal fibula's great positional variability (15, 18) and on its ossification stage (19). Using the TA enables us to overcome this anatomical variability while reproducing the tibiotalar joint's geometric axis as closely as possible (20). The tibiotalar joint's geometric axis corresponds to a fixed axis about which the talus moves in the sagittal plane and gives a better appreciation of the foot's position during gait. In normal conditions, the talar axis and the forefoot axis are aligned; thus, we consider the talus’ orientation in the tibiofibular mortise is probably more important than the alignment of the malleolus when trying to appreciate the foot's position during gait. Our research underlined that the definition of the orientation of the distal tibia was probably the parameter requiring the most caution and around which the measurement discrepancies were concentrated. Tibial torsion angle was significantly lower when using the talar axis as the distal reference point (TA 17.2°** **±** **16.9°) than when using the intermalleolar axis (IMA 23.0°** **±** **16.2°) with a mean difference between the two TTA measurement techniques of 5.8°(*p*** **<** **0.001).

Finally, two-dimensional measurements of the TTA suffer from inaccuracies, with low inter- and intra-observer reliability between the methods reported (8–10, 18, 21). A new, three-dimensional measurement method was recently developed and seems to show high inter-observer reliability (15, 22). It can evaluate tibial torsion with or without considering the fibula's position. However, the technique seems complex, requiring the manual selection of multiple reference points. This complexity increases analysis time, human errors and, ultimately, misinterpretations. In addition, this method requires specific computer software, increasing costs and perhaps restricting its use in certain regions of the world.

Considering the present study's results, we recommend a two-dimensional CT imaging technique that requires no additional software and is reproducible in the daily clinical practice of most pediatric orthopedic surgeons. Measuring tibial torsion using the talar axis as a distal reference point showed excellent intra- and inter-observer reliability. In addition, TA measurement is significantly faster than IMA measurement, making it even more suitable for use in daily clinical practice.

This study has some limitations. Firstly, the CT scans used in this study were obtained with the patient lying supine with feet hold together. This position does not represent real life weight bearing conditions. It is known that hindfoot alignment significantly changes in the upright weight-bearing CT position in adults (23) and it would be interesting to implement this modality in further studies. In this work, only indirect measurements of tibial torsion are considered. Furthermore, these measurements cannot be correlated with clinical and functional outcomes. It would be interesting to do quantitative instrumented gait analysis to correlate CT scan measurement, tibial torsion and foot progression angle (24).

In addition, this study is only focused in children without neurologic or syndromic pathologies. We consider that the technique should be validated first in “healthy” patients before exporting it to patients who are known to develop epiphyseal dysmorphia such as cerebral palsy. This will require further studies to accurately define the axes of the proximal and the distal tibia in children with skeletal morphological issues to verify the applicability of the proposed method.

Finally, this alternative method needs to be validated through multi-center studies using different CT models.

## 5 Conclusion

Tibial torsion values may differ significantly depending on the axis chosen to define tibial orientation. In this study, at the level of the proximal tibia, choosing the proximal transtibial angle (PTTA) or the posterior margin tibial plateau angle (PMTPA) had little influence on the value of the tibial torsion angle. However, PMTPA was faster (36** **±** **4 s vs. 144** **±** **11 s for PTTA, *p*** **<** **0.001). At the level of the distal tibia, there was a significant difference of 5.8° between intermalleolar angle (IMA: 23.0°** **±** **16.2°) and talar angle (TA: 17.2°** **±** **16.9°, *p*** **<** **0.001). However, TA was faster (72** **±** **7 s vs. 134** **±** **15 s for IMA, *p*** **<** **0.001) and using TA frees the clinician of distal fibula's great positional and ossification variability in the pediatric population. Thus, using the PMTPA and TA could be more suited to daily clinical practice because tracing them is simple and fast, with high intra- and inter-observer reliability. The clinical relevance of this measurements and their feasibility in neurologic or syndromic children remains to be proven in further studies.

## Data Availability

The raw data supporting the conclusions of this article will be made available by the authors, without undue reservation.

## References

[1] Grisch D Dreher T. Torsion and torsional development of the lower extremities. Orthopade. (2019) 48(6):523–30. 10.1007/s00132-019-03752-3 31089774

[2] Schwartz M Lakin G. The effect of tibial torsion on the dynamic function of the soleus during gait. Gait Posture. (2003) 17(2):113–8. 10.1016/S0966-6362(02)00058-9 12633770

[3] Hicks J Arnold A Anderson F Schwartz M Delp S. The effect of excessive tibial torsion on the capacity of muscles to extend the hip and knee during single-limb stance. Gait Posture. (2007) 26(4):546–52. 10.1016/j.gaitpost.2006.12.003 17229573 PMC2443695

[4] Cibulka MT Winters K Kampwerth T McAfee B Payne L Roeckenhaus T Predicting foot progression angle during gait using two clinical measures in healthy adults, a preliminary study. Int J Sports Phys Ther. (2016) 11(3):400–8. 27274426 PMC4886808

[5] Bruderer-Hofstetter M Fenner V Payne E Zdenek K Klima H Wegener R. Gait deviations and compensations in pediatric patients with increased femoral torsion. J Orthop Res Off Publ Orthop Res Soc. (2015) 33(2):155–62. 10.1002/jor.22746 25284013

[6] Basaran SH Ercin E Bayrak A Cumen H Bilgili MG Inci E The measurement of tibial torsion by magnetic resonance imaging in children: the comparison of three different methods. Eur J Orthop Surg Traumatol Orthop Traumatol. (2015) 25(8):1327–32. 10.1007/s00590-015-1694-2 26325249

[7] Tomczak RJ Guenther KP Rieber A Mergo P Ros PR Brambs HJ. MR imaging measurement of the femoral antetorsional angle as a new technique: comparison with CT in children and adults. AJR Am J Roentgenol. (1997) 168(3):791–4. 10.2214/ajr.168.3.9057536 9057536

[8] Liodakis E Doxastaki I Chu K Krettek C Gaulke R Citak M Reliability of the assessment of lower limb torsion using computed tomography: analysis of five different techniques. Skeletal Radiol. (2012) 41(3):305–11. 10.1007/s00256-011-1185-4 21560009

[9] Jakob R Haertel M Stussi E. Tibial torsion calculated by computerised tomography and compared to other methods of measurement. J Bone Joint Surg Br. (1980) 62-B(2):238–42. 10.1302/0301-620X.62B2.7364840 7364840

[10] Jend HH Heller M Dallek M Schoettle H. Measurement of tibial torsion by computer tomography. Acta Radiol Diagn (Stockh). (1981) 22(3A):271–6. 10.1177/028418518102203A10 7315504

[11] Reikerås O Høiseth A. Torsion of the leg determined by computed tomography. Acta Orthop Scand. (1989) 60(3):330–3. 10.3109/17453678909149288 2750510

[12] Gruskay JA Fragomen AT Rozbruch SR. Idiopathic rotational abnormalities of the lower extremities in children and adults. JBJS Rev. (2019) 7(1):e3. 10.2106/JBJS.RVW.18.00016 30624306

[13] Güven M Akman B Unay K Ozturan EK Cakici H Eren A. A new radiographic measurement method for evaluation of tibial torsion: a pilot study in adults. Clin Orthop. (2009) 467(7):1807–12. 10.1007/s11999-008-0655-z 19052824 PMC2690742

[14] Claassen L Luedtke P Yao D Ettinger S Daniilidis K Nowakowski AM Ankle morphometry based on computerized tomography. Foot Ankle Surg Off J Eur Soc Foot Ankle Surg. (2019) 25(5):674–8. 10.1016/j.fas.2018.08.002 30306892

[15] Hoch A Roth T Marcon M Fürnstahl P Fucentese SF Sutter R. Tibial torsion analysis in computed tomography: development and validation of a real 3D measurement technique. Insights Imaging. (2021) 12(1):18. 10.1186/s13244-020-00960-w 33587196 PMC7884516

[16] Stephen JM Teitge RA Williams A Calder JDF El Daou H. A validated, automated, 3-dimensional method to reliably measure tibial torsion. Am J Sports Med. (2021) 49(3):747–56. 10.1177/0363546520986873 33533633 PMC7917570

[17] Tamari K Tinley P Briffa K Breidahl W. Validity and reliability of existing and modified clinical methods of measuring femoral and tibiofibular torsion in healthy subjects: use of different reference axes may improve reliability. Clin Anat. (2005) 18(1):46–55. 10.1002/ca.20050 15597368

[18] Madadi F Madadi F Maleki A Shamie AN Washington ER Yazdanshenas H. A new method for tibial torsion measurement by computerized tomography. J Orthop. (2016) 13(1):43–7. 10.1016/j.jor.2015.09.001 26955223 PMC4761617

[19] McDermott JE Scranton PE Rogers JV. Variations in fibular position, talar length, and anterior talofibular ligament length. Foot Ankle Int. (2004) 25(9):625–9. 10.1177/107110070402500905 15563383

[20] Yu J Cao S Wang C Zhao D Wang S Zhang C *In vivo* evaluation of the position and orientation of the geometric axis of the tibiotalar joint. Rong Q, éditeur. Appl Bionics Biomech. (2023) 2023:1–7. 10.1155/2023/2763099 PMC9876701 36713625

[21] Goutallier D Van Driessche S Manicom O Sariali E Bernageau J Radier C. Influence of lower-limb torsion on long-term outcomes of tibial valgus osteotomy for medial compartment knee osteoarthritis. J Bone Joint Surg Am. (2006) 88(11):2439–47. 10.2106/JBJS.E.01130 17079402

[22] Shin SY Yoon CH Lee ES Oh MK Kim AR Park JM The availability of radiological measurement of tibial torsion: three-dimensional computed tomography reconstruction. Ann Rehabil Med. (2011) 35(5):673–9. 10.5535/arm.2011.35.5.673 22506190 PMC3309262

[23] Hirschmann A Pfirrmann CWA Klammer G Espinosa N Buck FM. Upright cone CT of the hindfoot: comparison of the non-weight-bearing with the upright weight-bearing position. Eur Radiol. (2014) 24(3):553–8. 10.1007/s00330-013-3028-2 24071992

[24] Davids JR Davis RB Jameson LC Westberry DE Hardin JW. Surgical management of persistent intoeing gait due to increased internal tibial torsion in children. J Pediatr Orthop. (2014) 34(4):467–73. 10.1097/BPO.0000000000000173 24531409

